# Supplementary material for: A New Family of Transcriptional Regulators Activating Biosynthetic Gene Clusters for Secondary Metabolites
Source: Int J Mol Sci. 2022 Feb 23;23(5):2455. doi: 10.3390/ijms23052455 (PMC8910723; doi:10.3390/ijms23052455)
Supplement: Supplementary file 1 [file ijms-23-02455-s001.zip › ijms-1521819-supplementary.pdf]

Supplementary Materials

International Journal of Molecular Sciences; section: Molecular Microbiology; special issue: Bacterial Regulatory Proteins

A new family of transcriptional regulators activating biosynthetic gene clusters for secondary metabolites

Renata Novakova, Erik Mingyar, Lubomira Feckova, Dagmar Homerova, Dominika Csolleiova, Bronislava Rezuchova, Beatrica Sevcikova, Rachel Javorova, Jan Kormanec*

Institute of Molecular Biology, Slovak Academy of Sciences, 845 51 Bratislava, Slovak Republic.

* Correspondence and reprints: jan.kormanec@savba.sk, Tel.: +421 (2) 59307419,
ORCID ID: 0000-0003-0377-6413

Table of contents Page

Figure S1: Comparison of Aur1P with similar atypical response regulators from the BGCs 2
Figure S2: A comparison of Aur1O with similar proteins from unknown BGCs 4
Table S1: Oligonucleotides used in this study. 5

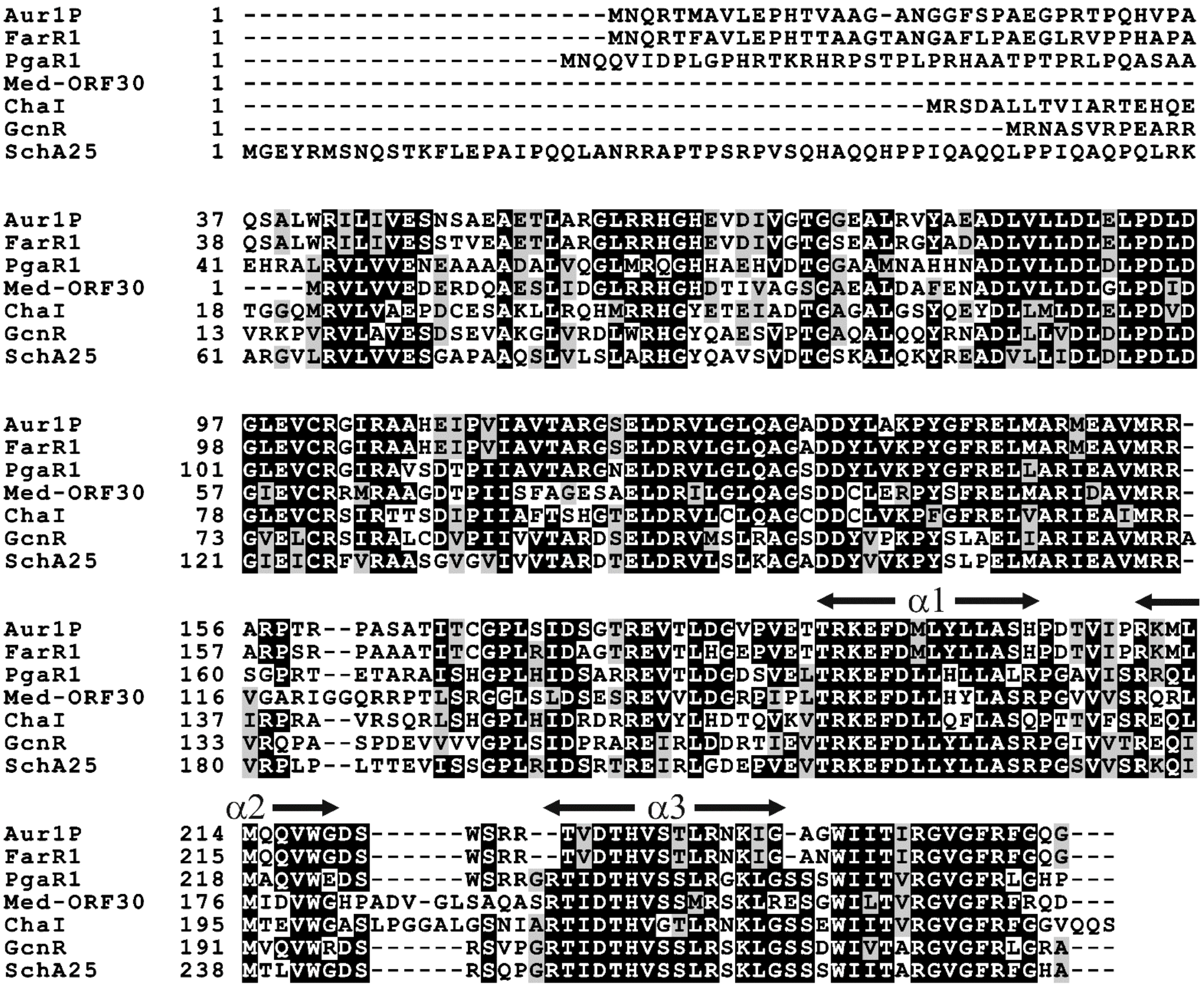

Figure S1. Comparison of Aur1P with similar atypical response regulators (ARRs) from the BGCs containing the *aur1O* homologous gene (Figure 6). Protein sequences and accession numbers are: Aur1P of *S. lavendulae* subsp. *lavendulae* CCM 3239 (AAX57187), FarR1 of *S. lavendulae* FRI-5 (BAG74710), PgaR1 of *Streptomyces* sp. PGA64 (AHW57766), Med-ORF30 of *Streptomyces* sp. AM-7161 (BAC79018), ChaI of *S. chattanoogensis* L10 (AIU99192), GcnR of *S. lusitanus* (AGO50631), SchA25 of *Streptomyces* sp. SCC-2136 (CAH10125). Identical residues are highlighted in black. Similar residues are shaded. The location of the secondary structure helical elements of homologous OmpR (Novakova et al. 2005) of the DNA-binding effector domain determined from its tertiary structure (Martinez-Hackert and Stock, 1997) are shown above the sequence. The numbers refer to the deposited amino acid sequences in databases.

References

Martinez-Hackert, E; Stock, A.M. Structural relationships in the OmpR family of winged-helix transcription factors. *J. Mol. Biol.* **1997,** *269,* 301-312.

Novakova, R.; Homerova, D.; Feckova, L.; Kormanec, J. Characterization of a regulatory gene essential for the production of the angucycline-like polyketide antibiotic auricin in *Streptomyces aureofaciens* CCM 3239. *Microbiology-SGM* **2005,** *151,* 2693-2706.

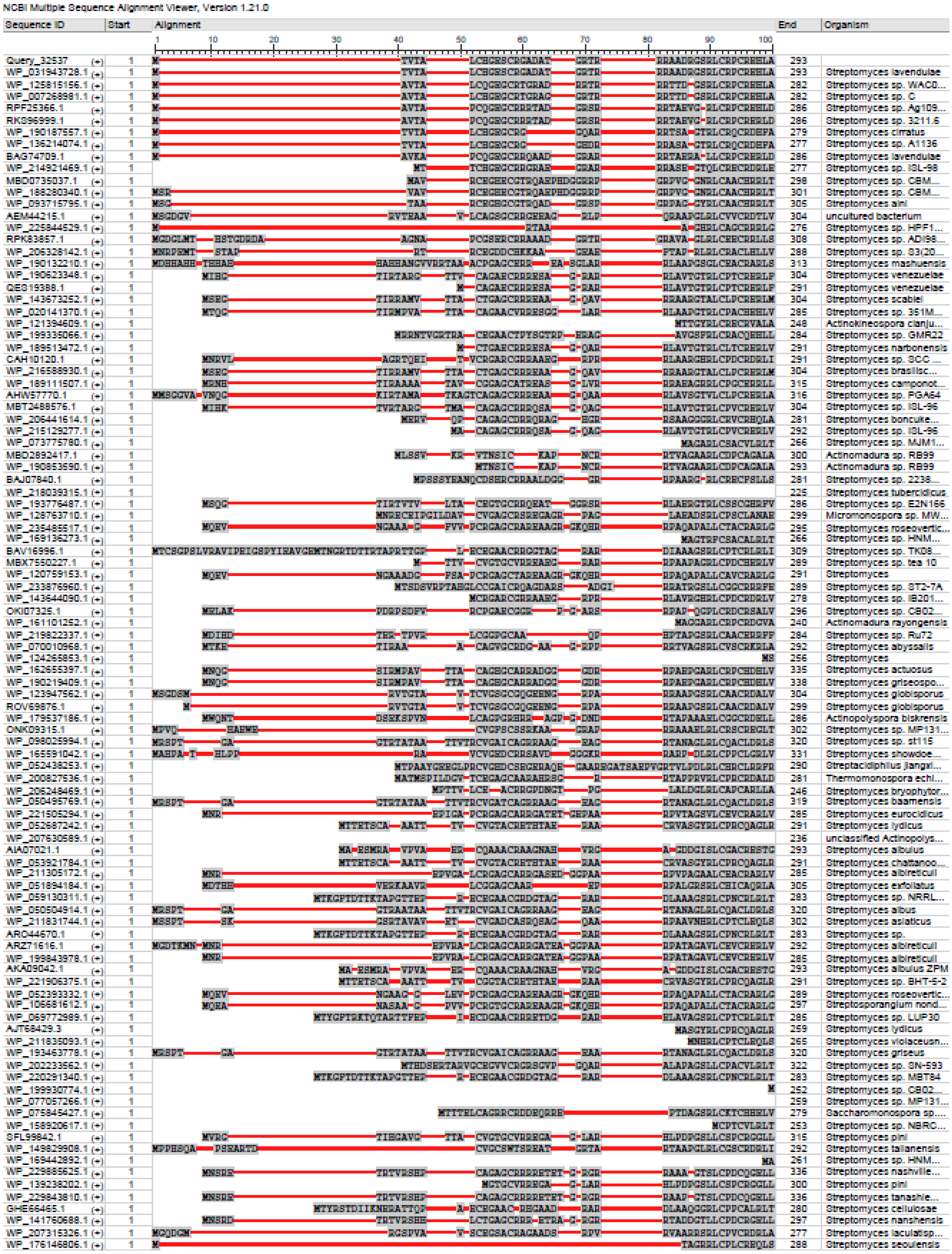
NCBI Multiple Sequence Alignment Viewer, Version 1.21.0
Sequence ID
Start
Alignment
End
Organism

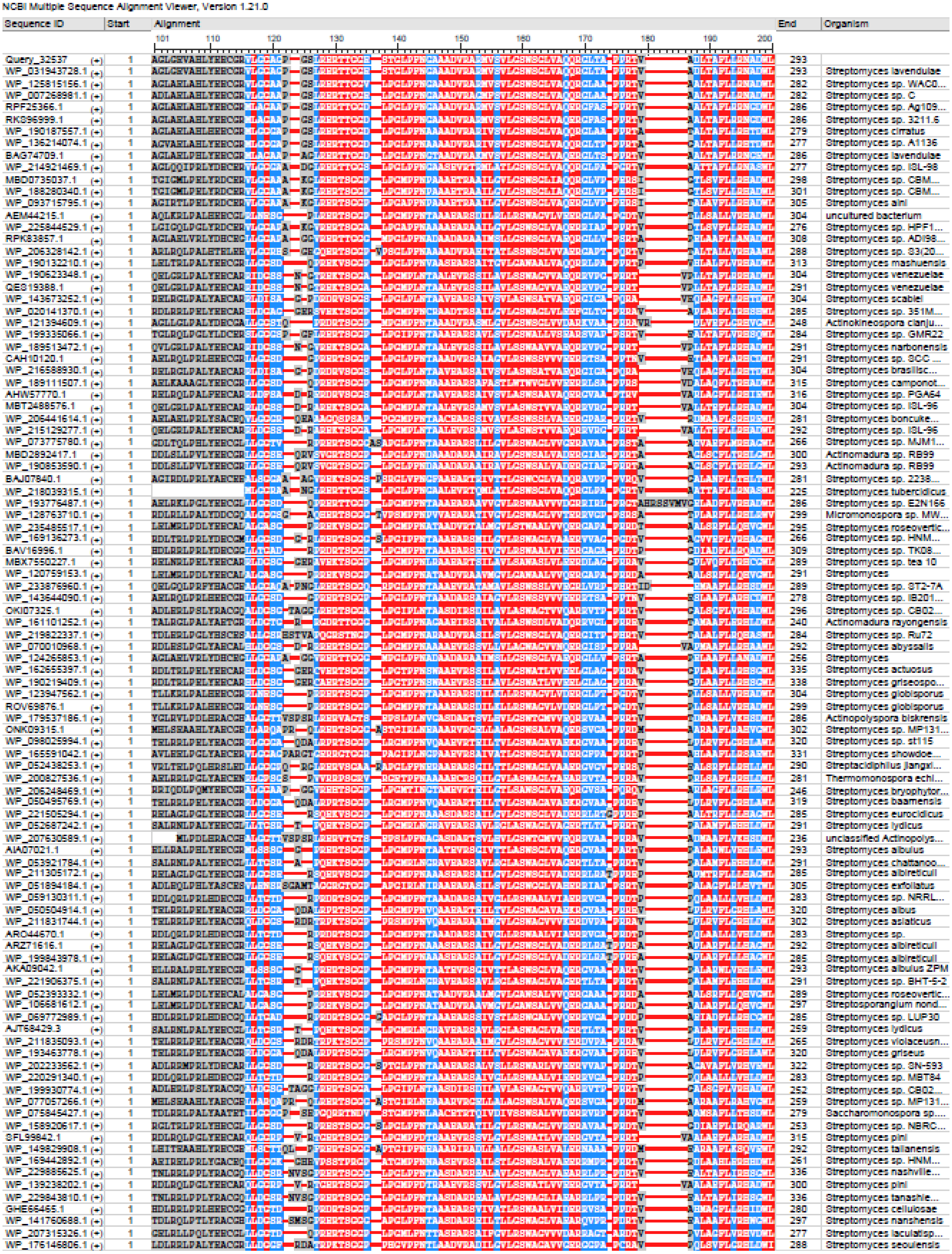
NCBI Multiple Sequence Alignment Viewer, Version 1.21.0
Sequence ID
Start
Alignment
End
Organism

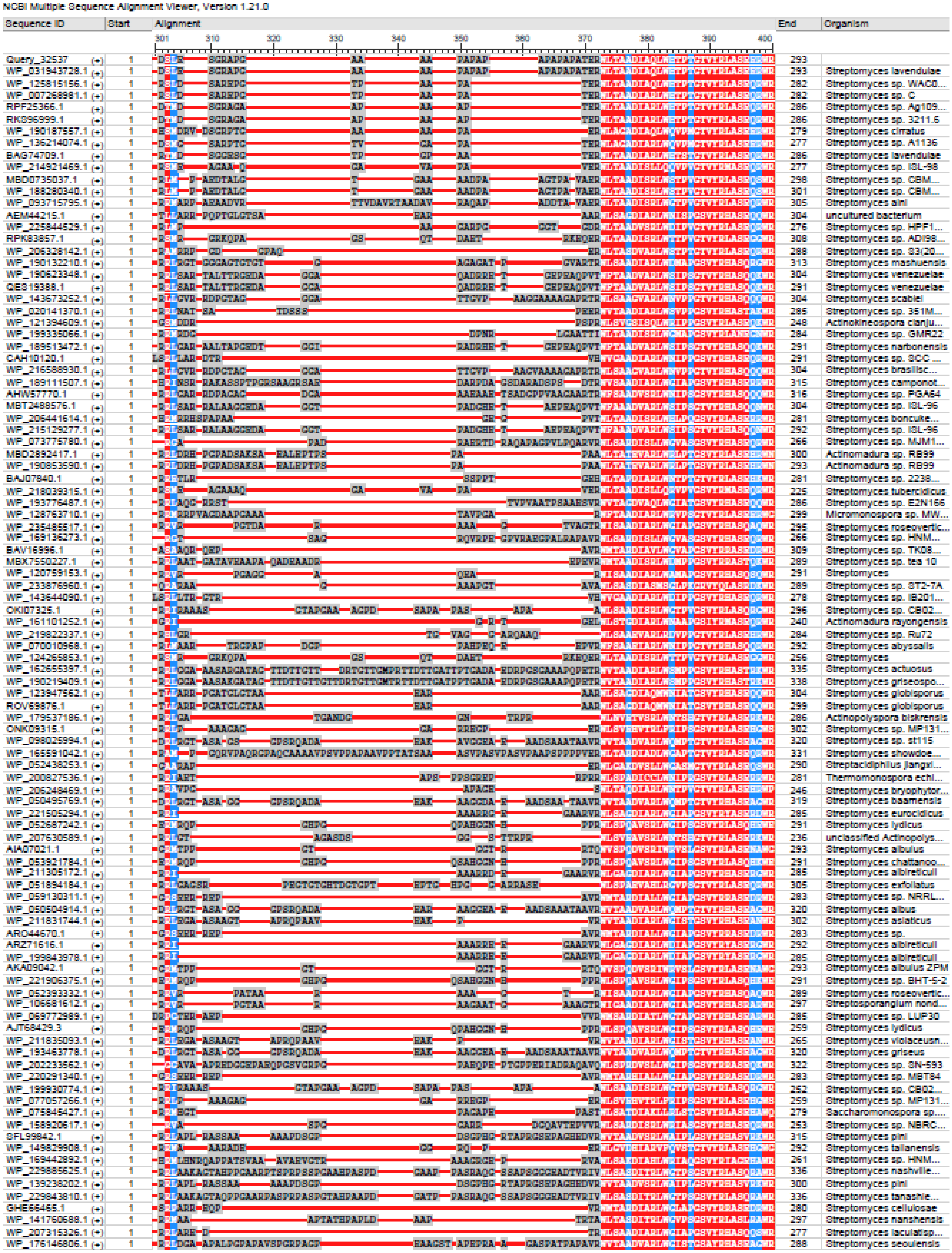
NCBI Multiple Sequence Alignment Viewer, Version 1.21.0
Sequence ID
Start
Alignment
End
Organism

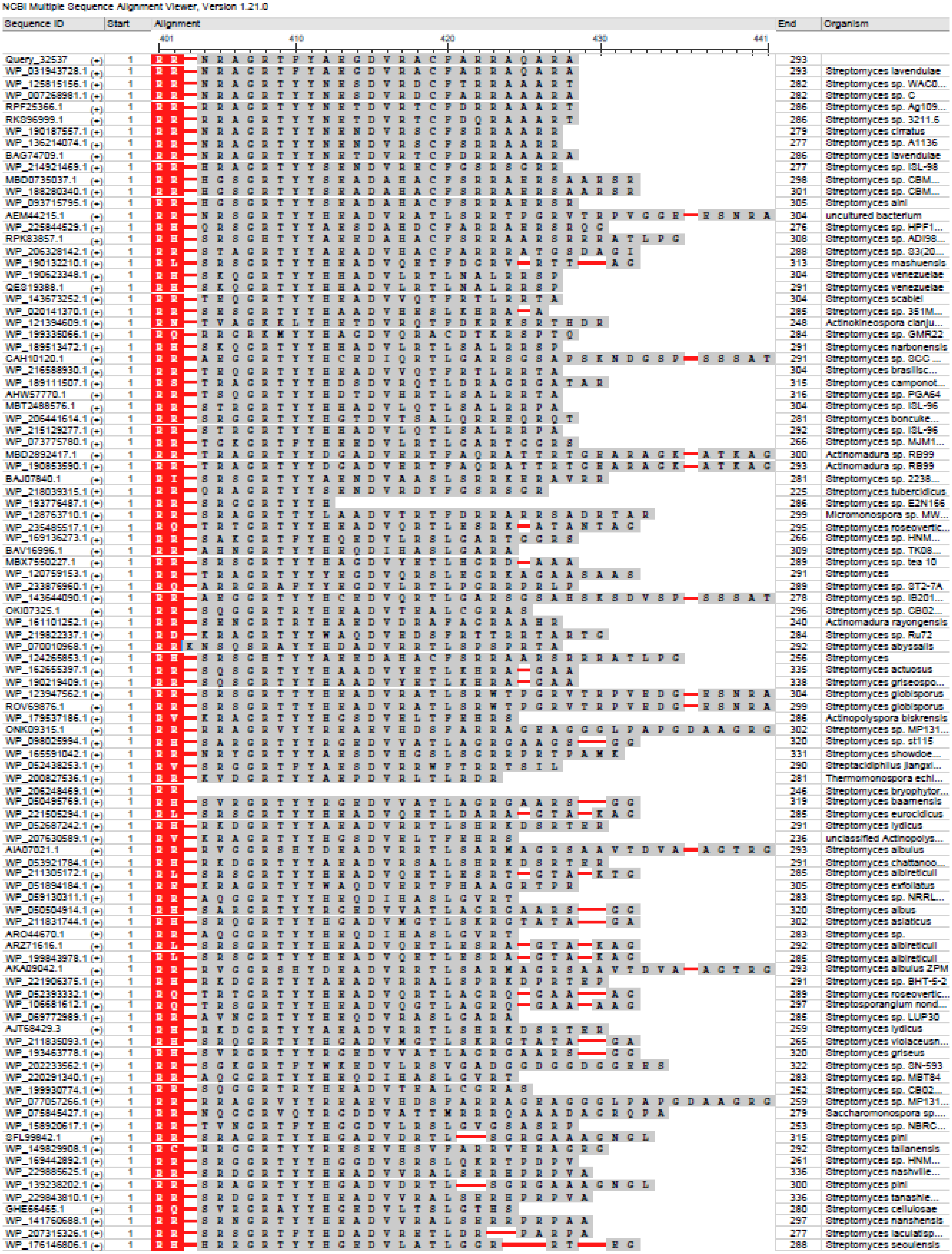
NCBI Multiple Sequence Alignment Viewer, Version 1.21.0
Sequence ID
Start
Alignment
End
Organism

Figure S2. Comparison of Aur1O with similar proteins from unknown BGCs. Protein sequences and accession numbers are in the left column. Aur1O from *S. lavendulae* subsp. *lavendulae* CCM 3239 (AAK59995) is referred to as Querry_32537. The Aur1O amino acid sequence comparison with the non-redundant protein database was performed using a BlastP browser and NCBI multiple sequence alignment viewer (https://blast.ncbi.nlm.nih.gov/). The numbers refer to the deposited amino acid sequences in databases.

Table S1. Oligonucleotides used in this study. Cloning sites are underlined.

Oligonucleotide	Sequence (5′ − 3′)
aur1AFw	CGCAGTCCGTCACCGAGACCC
aur1ARv	CGTCGTAGGTGATCTCCAGTCC
aur1OdDir	CCGACCAGCGACCACAGACACGAGCCGAGGGGGAACGACATTCCGGGGATCCGTCGACC
aur1OdRev	CCTGCGCCCTGCGGGCGAAGCAGGCGCGGACGTCGCCCTCTGTAGGCTGGAGCTGCTTC
aur1OCla	CCCCCATCGATCCGAGGGGGAACGACATGACG
aur1OXba	CCCCCTCTAGAGCTCAGGCGCGGGCCTGCGCCC
aur1AS1dir	CGTGGCCTTGACACTCCCTTGGC
aur1AS1rev	CGCCGCTCCAGACGTTCGAGGACG
aur1ApDir	CCCCGGATCCCAATTGCCGCTAGCCCCTTAAGCCATTAATCCTTCGAAGGACCCGCACAGCAACCGGTAGG
aur1ApRev	CCCCGGTACCCGCCTCCGGAGACAGTGCACCG
aur1PpDir	CCCCGATATCCCCGATTTAACCCTTTTATCGGC
aur1PpRev	CCCCTTCGAAGGTCCGGCCGGGCTCAGCCCTGG
aur1OpRev	CCCCTTCGAAGGGAACGTCGTGCGGAAGACCGG
luxCrev	GCTCTCGGGGAAGATCTCGACCTGG
pMU1fd	CGGACGGTTCGGCCCCTTTTTTGG
aur1PDHdir	GGGGTCTAGACCATATGAACCAGCGGACCATGGCTGTGC
aur1PDHrev	GGGGGTACCTCGAGTCAGCCCTGGCCGAAGCGGAATCC
aur1ODHdir	GGGGTCTAGACCATATGACGGTGACCGCGCTGTGCCACG
aur1ODHrev	GGGGGTACCTCGAGTCAGGCGCGGGCCTGCGCCCTGC
KT25dir	GCCATTATGCCGCATCTGTCC
KT25rev	GCGATTAAGTTGGGTAACGCC
UT18Cdir	GCGTGCCGAGCGGACGTTCG
UT18Crev	CGGGGCTGGCTTAACTATGC
